# Intercolonial Microdamage and Cracking Micromechanisms during Wire Drawing of Pearlitic Steel

**DOI:** 10.3390/ma16051822

**Published:** 2023-02-22

**Authors:** Jesús Toribio, Francisco-Javier Ayaso, Rocío Rodríguez

**Affiliations:** Fracture & Structural Integrity Research Group (FSIRG), University of Salamanca (USAL), E.P.S., Campus Viriato, Avda. Requejo 33, 49022 Zamora, Spain

**Keywords:** high-strength pearlitic steel, cold-drawing, pearlite colonies, ferrite/cementite lamellae, drawing-induced micro-defects, intercolonial microdamage (ICMD), cracking mechanisms

## Abstract

This paper studies the drawing-induced intercolonial microdamage (ICMD) in pearlitic microstructures. The analysis was performed from the direct observation of the microstructure of the progressively cold-drawn pearlitic steel wires associated with the distinct steps (*cold-drawing passes*) of a real cold-drawing manufacturing scheme, constituted by seven cold-drawing passes. Three types of ICMD were found in the pearlitic steel microstructures, all affecting two or more pearlite colonies, namely: (i) *intercolonial tearing*; (ii) *multi-colonial tearing*; and (iii) *micro-decolonization*. The ICMD evolution is quite relevant to the subsequent fracture process of cold-drawn pearlitic steel wires, since the drawing-induced intercolonial micro-defects act as weakest links or fracture promoters/initiators, thereby affecting the microstructural integrity of the wires.

## 1. Introduction

High-strength cold-drawn eutectoid pearlitic steels are used in wire form in civil engineering as the main constituents of prestressed concrete structures, so that commercially they are well known as prestressing steel wires [1,2] and can be considered as high-performance materials [2,3,4] with an extremely high strength only limited by the appearance of cleavage fracture.

From the point of view of mechanical behaviour of the material, the cold-drawing process generates an increase of yield stress and ultimate tensile strength of the steel by means of a strain hardening mechanism. Many scientific papers analyzed the microstructural evolution in pearlitic steels during cold drawing and its relationship with strength and ductility, from the pioneering analyses carried out by Embury and Fisher [5] and Langford [6] about drawing and deformation of pearlite, to the review of data on the interlamellar spacing paper by Ridley [7] or the study of the important role of the prior austenite grain by Lewandowski and Thompson [8]. It is worth mentioning the key studies during the 1990s by Nam and Bae [9], Read et al. [10], Toribio and Ovejero [11,12,13,14], Languillaume [15], and Nam et al. [16], mostly dealing with microstructural evolution of pearlite by cold drawing [9,10,11,12,13,14] and the latter [15,16] dealing with the specific phenomenon of drawing-induced cementite dissolution.

The primary objective of cold drawing as a manufacturing process based on a strain hardening mechanism is the strengthening of the material to be used in engineering for high performance purposes. Many papers in the scientific literature have dealt with the plasticity (yielding) behavior, by analyzing the Hall—Petch parameter [17,18,19,20], although the relationship between microstructure and strength in the case of pearlite sometimes does *not* fit a Hall—Petch equation [19] but more properly an Embury—Fisher law [20]. A wide range of scientific papers have dealt with the study of different hardening and strengthening mechanisms after the cold drawing of pearlitic microstructures [21,22,23,24,25,26,27,28].

Other scientific references analyze the fracture (post-yield) mechanical behavior of pearlitic microstructures, from the pioneering articles written by Porter et al. [29], Hyzak and Bernstein [30], Park and Bernstein [31], Alexander and Bernstein [32], and Dollar et al. [33], to more recent ones [34,35,36,37,38,39] dealing with yielding and fracture behavior.

In addition to the aforesaid primary objective of drawing-induced strength increase, some secondary side effects appear during manufacturing. First of all, anisotropy appears after heavy drawing in the matter of yielding and plasticity (anisotropic plastic behaviour), as reported by Toribio et al. [40], and also linked with fatigue and fracture performance (anisotropic fatigue & fracture behaviour), as described elsewhere [41,42,43,44,45,46,47,48,49] with the result of mixed-mode fracture propagation and strength anisotropy. In the matter of the relevant fractography linked to the aforesaid anisotropic performance, a non-conventional type of cleavage has been reported by Toribio and Ayaso [50], and the innovative concept of exfoliation fracture has been used [51], as well as the idea of delamination cracks [52,53,54,55].

In this paper, the main cracking micromechanisms in cold-drawn eutectoid pearlitic steels are discussed on the basis of the intercolonial microdamage (ICMD) appearing in the pearlitic steel wires during the cold-drawing manufacture procedure. On the basis of the aforesaid existing studies (research background), specific analysis of the evolution with cold drawing of ICMD in the important case of pearlitic microstructures has not been found in the scientific literature, so the present paper offers a novel approach. Thus, the role of the manufacturing process by cold drawing in the fracture behavior of prestressing steel in service is clarified, thereby providing insight into the effect of manufacturing by cold drawing in the fracture performance and macro- and micro-structural integrity of prestressed concrete structures used in civil engineering.

## 2. Experimental Procedure

### 2.1. Materials

A pearlitic steel taken from a real (industrial) wire drawing chain, constituted by seven steps of cold drawing, was used in the present research paper. It was analyzed from the initial hot rolled bar (not cold-drawn at all) to the final commercial prestressing wire (heavily cold-drawn in seven steps). To identify the wires, the nomenclature consists of a letter to represent the wire family and a number indicating the drawing passes undergone by the specific wire: 0 for the initial wire rod, 7 for the commercial product, and numbers from 1 to 6 for the wires taken from the intermediate stages. The chemical composition of steel family E is given in Table 1 (more details in Ref. [34]).

**Figure 1:**
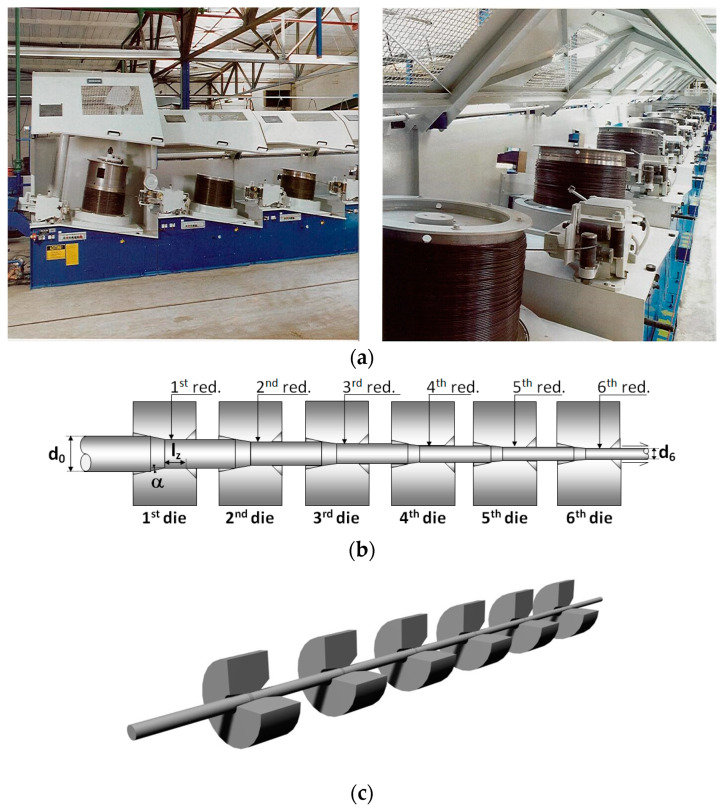
Manufacture of prestressing wires by progressive cold drawing: (**a**) two photographs of a real (in situ) cold drawing procedure in the factory; (**b**) scheme of a typical drawing process with six passes; (**c**) sketch of a 3D view of such a process.

The manufacture of commercial prestressing steel wire for prestressed concrete is made in the form of progressive (multi-pass) cold drawing of a previously hot-rolled bar with pearlitic microstructure. Figure 1a shows two views of the real cold-drawing process in the factory. Figure 1b draws a typical drawing process consisting of six passes through the corresponding dies (with their associated diameter reductions), and finally Figure 1c sketches a 3D view of the stages of passing through the drawing dies.

**Figure 2:**
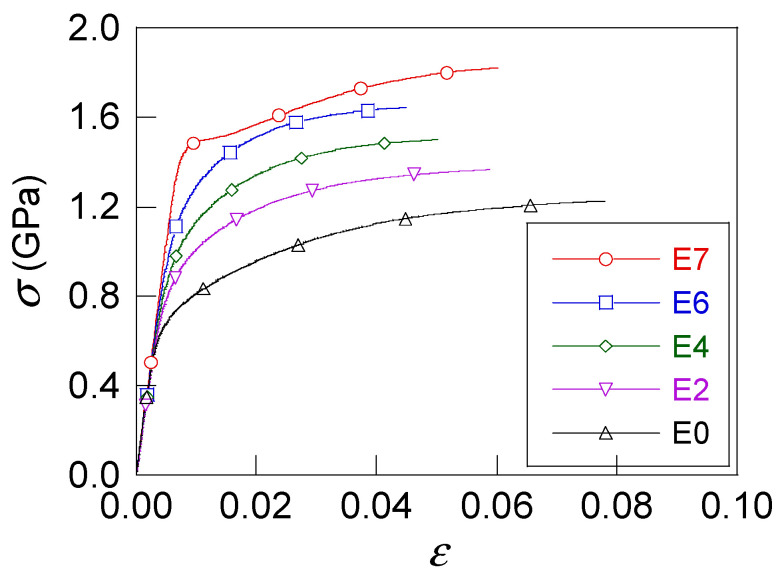
True stress vs. true strain (*σ*-*ε*) of steels from family E.

In the present article a special manufacture row consisting of seven drawing passes (one more than in the previous figure associated with a quite conventional procedure) was considered and the following wires were used: initial hot rolled bar E0 (not cold-drawn at all) and the wires from the second, fourth, sixth and seventh drawing passes (E2, E4, E6 and E7, respectively) of the drawing process (slightly drawn steels, E2 and E4; heavily drawn steels, E6 and E7). Table 2 gives the diameter (*D*), the cumulative plastic strain at the end of each drawing stage (*ε*^P^_cum_), Young’s modulus (*E*), the yield stress (*σ*_Y_), the ultimate tensile stress (*σ*_R_), and the ductility (the percent of reduction in area %*RA*) for each wire. Figure 2 shows the true stress vs. true strain curves (*σ-ε*) for all steels. Tensile tests were performed using a 50 mm gauge length and a constant displacement rate (crosshead speed) of 2 mm/min) in the testing machine, as described elsewhere [34].

### 2.2. Methods

**Figure 3:**
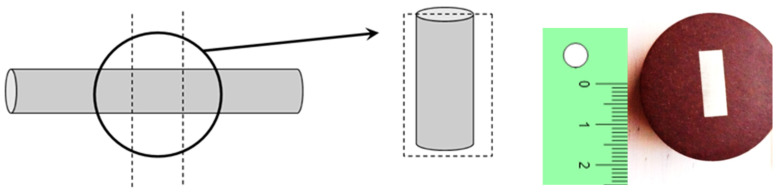
Preparation of pearlitic steel samples: longitudinal cutting (**left**); mounted in resin (**right**).

The metallographic (or materialographic) study was performed after longitudinal cuts (sections) in the wires associated with the different drawing passes, as shown in Figure 3 (left). In this paper the micrographs are always oriented with their vertical side parallel to the drawing direction (or longitudinal wire axis). The longitudinal sections were carefully prepared to obtain a good materialographic view in the scanning electron microscope (SEM). To prepare the micrographs, the described sections were embedded in a phenolic resin and thermally hardened by hot compression assembly, as depicted in Figure 3 (right).

A further phase is the mechanical preparation, i.e., the grinding and polishing of the samples, to achieve a specular surface on which a chemical attack can be made, thereby revealing the microstructure of the corresponding steel wire. Such a chemical attack was made with nitric acid (HNO_3_) to 3% of solution in pure commercial ethanol. The samples were analysed by a scanning electron microscopy using a JEOL JSM-5610 LV (Jeol Ltd., Tokyo, Japan).

## 3. Microstructural Evolution during Cold Drawing of Pearlite

The hierarchical microstructural evolution in pearlitic steels during cold drawing (at the two microstructural levels of pearlitic colonies and lamellae) was studied in the pioneering papers by Toribio and Ovejero [11,12,13,14] showing two fundamental trends: the slenderizing of the pearlite colonies [11], the increase in packing closeness associated with the decrease in interlamellar spacing [12] and orientation (alignment) in the direction of cold drawing (wire axis) of both the pearlitic colonies and lamellae [13,14].

**Figure 4:**
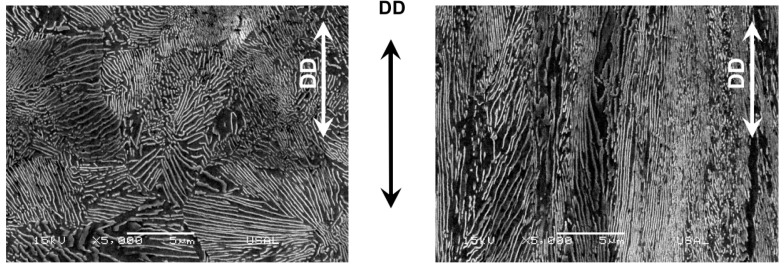
Longitudinal metallographic section of a hot rolled bar E0 (**left**) and a cold-drawn wire E7 (**right**).

Figure 4 shows the two microstructural levels (pearlite colonies and Fe/Fe_3_C lamellae, the former being domains in which the latter have common orientation) at the initial and final stages of microstructural evolution after cold drawing, i.e., the two micrographs correspond to a hot rolled bar (left: ‘randomly oriented’, since it is not drawn at all) and a heavily drawn pearlitic steel wire or commercial prestressing steel (right: ‘markedly oriented’ in the drawing direction, DD, after heavy drawing).

## 4. Microstructural Damage Analysis and Consequences on Cracking and Fracture

### 4.1. Intercolonial Microdamage (ICMD)

As explained in the previous section in the matter of the pearlite colony or first microstructural level, Toribio and Ovejero [13] showed that such colonies become progressively oriented during the manufacture by strain hardening, so that they *do* rotate during the heavy cold drawing process, with the possibility of certain microdamage as a consequence of lateral constraint imposed by the drawing dies and very severe plastic deformation. This section analyzes this kind of microdamage related to colony boundaries, i.e., the ‘intercolonial microdamage’. Three types of damage have been found, all affecting two or more pearlitic colonies, namely: (i) intercolonial tearing; (ii) multi-colonial tearing; and (iii) micro-decolonization.

**Figure 5:**
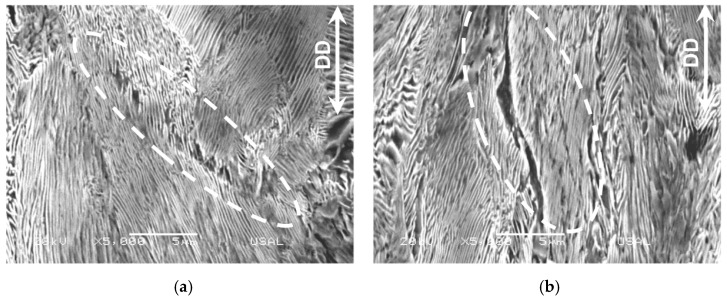
Intercolonial tearing at the boundary of two adjacent pearlite colonies: (**a**) in a slightly drawn pearlitic steel after two drawing passes; (**b**) in a heavily drawn pearlitic steel after six drawing passes.

The first type, the (i) intercolonial tearing takes place at any cold-drawing degree by friction-induced local tearing (therefore, a kind of drawing-induced local tearing) at the boundary between two adjacent pearlitic colonies that are in close contact during cold drawing and suffer rotation of the colonies themselves. This intercolonial tearing is a sort of localized damage (or microdamage) represented in the Figure 5a for a slightly drawn pearlitic steel and in Figure 5b for a heavily drawn pearlitic steel after six passes through the drawing dies. It is seen that this microdamage (a sort of *single* micro-crack) rotates and orientates in the drawing direction (DD) as the drawing degree increases, and the same happens to the pearlitic colonies themselves that become progressively oriented along the DD, cf. [13].

**Figure 6:**
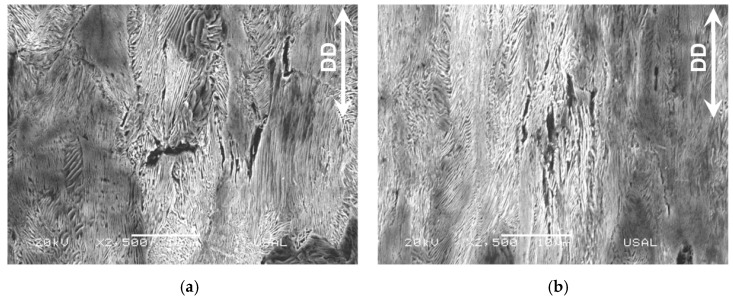
Multicolonial tearing of several neighboring pearlite colonies: (**a**) in a slightly drawn pearlitic steel after two drawing passes; (**b**) in a heavily drawn pearlitic steel after six drawing passes.

**Figure 7:**
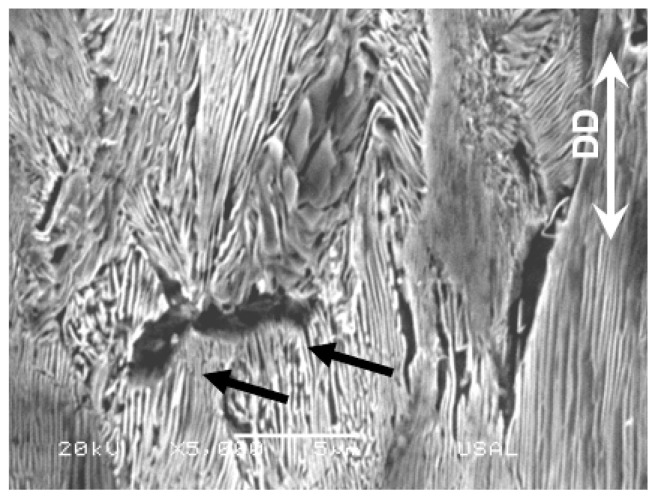
Higher magnification of multicolonial tearing of several neighboring pearlite colonies in a slightly drawn pearlitic steel after two drawing passes.

The second type, (ii), of intercolonial microdamage, i.e., the multicolonial tearing takes place also at the boundary between colonies (as the type I intercolonial tearing), but in this case the microdamage is not so localized in a single line of boundary (a crack in the section of the micrograph), but extended in volume (an area in the section representing the micrograph) and affecting a set of different neighboring colonies in the aforesaid volume (or area in the metallographic section). It mostly appears in pearlitic colonies containing ferrite/cementite lamellae markedly oriented in the DD (see Figure 6), either in slightly drawn steels (Figure 6a) or in heavily drawn steels (Figure 6b). Again, it is seen that this microdamage (a sort of multiple micro-cracking, i.e., a multi-cracking) mainly rotates and orientates predominantly in the drawing direction (DD) as the drawing degree increases and the pearlitic colonies containing them orientate, cf. [13]. In the slightly drawn pearlitic steels there is a single microcrack perpendicular to the DD (see black arrows in the enlarged view of Figure 7 with high magnification (×5000)), but in the heavily drawn pearlitic steel almost all micro-cracks are oriented along the DD.

**Figure 8:**
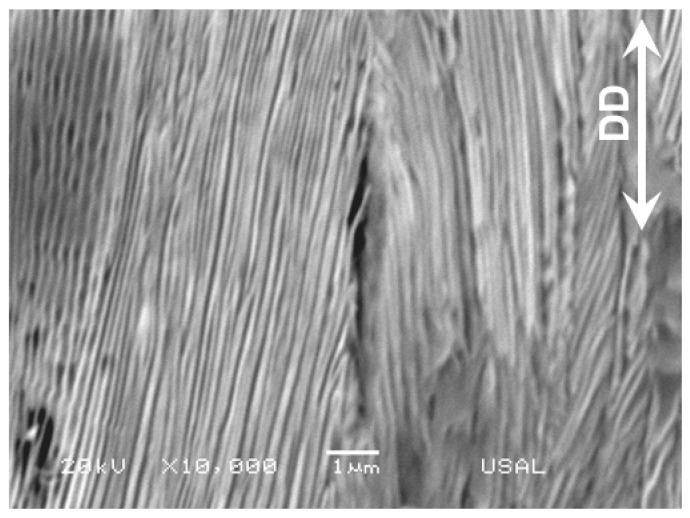
Micro-decolonization in a heavily drawn pearlitic steel (after six drawing stages).

The third type (iii) of intercolonial microdamage, i.e., the micro-decolonization, appears at the boundary between two adjacent colonies (like the type I intercolonial tearing), see Figure 7, but the mechanism of formation is just the opposite. Whereas the former (Figure 5) is caused by strong local compression, friction and tearing caused by the material passing through the drawing dies, the latter (Figure 8) is caused by some local tension during drawing organization of the material, thereby causing debonding between the two similar microstructural units, the pearlite colonies, i.e., provoking decolonization. It is a very localized damage associated with the final stages of cold drawing (i.e., for the most heavily drawn steels) and it is extended in a very small area requiring very high magnification. From the fracture mechanics viewpoint, it is a real micro-crack usually fully oriented quasi-parallel to the wire axis or DD. In Figure 8 it is seen that, in addition to the two adjacent colonies whose boundaries are fully oriented in the DD (together with the microdamage itself in the form of micro-decolonization), the ferrite and cementite lamellae inside the colonies are also fully oriented in such a DD.

### 4.2. Cracking Micromechanisms

With regard to cracking mechanisms in cold-drawn pearlitic steel, the three types of intercolonial microdamage represent different kinds of micro-cracks (or even groups of micro-cracks) more or less oriented in relation to the wire axis or DD (the higher the drawing degree, the more oriented the microdamage events in relation to the DD) that could affect the cracking micromechanisms and thus, the macroscopic fatigue and fracture behavior of the progressively cold-drawn pearlitic steels described in this paper. To analyze the different roles of intercolonial microdamage types in the cracking mechanisms, the three main cracking mechanisms in fracture mechanics will be analyzed, namely: (i) fatigue or subcritical cracking in air, (ii) critical (fracture) cracking in air, (iii) environmentally assisted cracking.

With regard to subcritical fatigue cracking (i), it was seen in previous works on this kind of steels [46], fatigue cracks are transcolonial and translamellar, i.e., they advance by crossing the pearlite colonies and breaking the ferrite/cementite lamellae, so that the colony boundary does not represent a critical issue in fatigue cracking, the key elements being the lamellae in the matter of orientation controlling the size and angle of micro-deflections in the fatigue crack path and the interlamellar spacing conditioning the free distance for dislocational movement in the ferritic phase.

**Figure 9:**
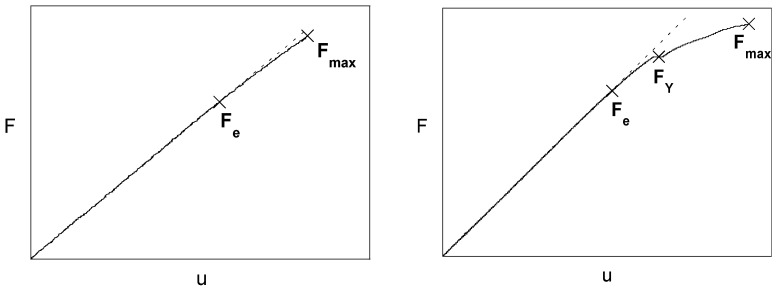
Load-displacement (*F*-*u)* plot: slightly drawn steel bar (**left**) and heavily drawn steel wire (**right**).

**Figure 10:**
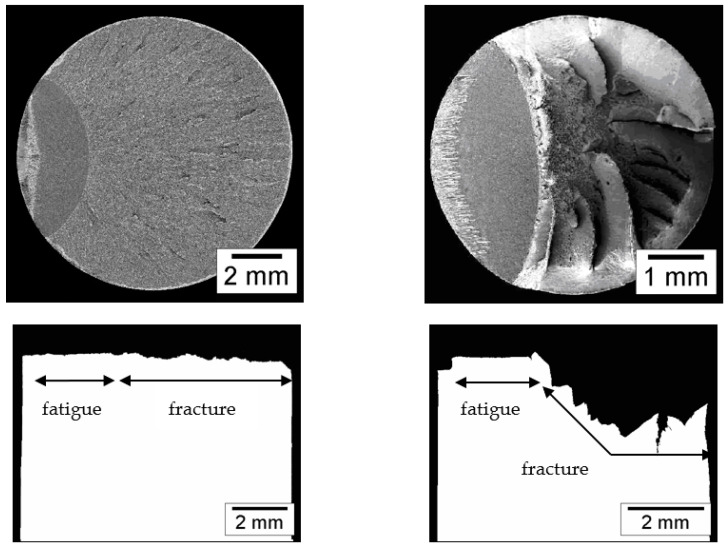
Fracture surface (**top**) and fracture profile (**bottom**) for a hot rolled or slightly drawn pearlitic steel bar (**left**) and for a heavily cold-drawn pearlitic steel wire (**right**).

In the matter of critical fracture/cracking (ii), it was seen previously [45,47] that the fracture/cracking behavior of the cold-drawn pearlitic steel becomes progressively anisotropic as the degree of cold drawing increases, with fracture/crack path deflection and mixed mode propagation, the deflection angle being also a rising function of the drawing degree. Figure 9 shows the load-displacement (*F-u*) plots in a fracture test on precracked samples of a slightly drawn and a heavily drawn pearlitic steel wire. The former is quasi-linear up to the final fracture, whereas the latter exhibit a load *F*_Y_ associated with a local instability (burst) or ‘pop-in’, after which the load still increases in a non-linear manner up to final fracture *F*_max_. Figure 10 shows the fracture surface and cracking/fracture profile for the slightly and heavily drawn pearlitic steels, where a stepped crack path profile is observed in the latter which exhibits strength anisotropy. The pop-in could be caused by any intercolonial microdamage, but probably the micro-decolonization could be the origin of the phenomenon. In addition, the small cracks produced by intercolonial tearing could produce crack deflection and the multi-cracking generated by multicolonial tearing could contribute to the link-up phase of microcracks in such an area of smeared damage in the form of multi-cracking generated at any pearlite colony boundary during drawing, thus creating the stepped cracking profile of Figure 10 (for more details see Ref. [47]).

**Figure 11:**
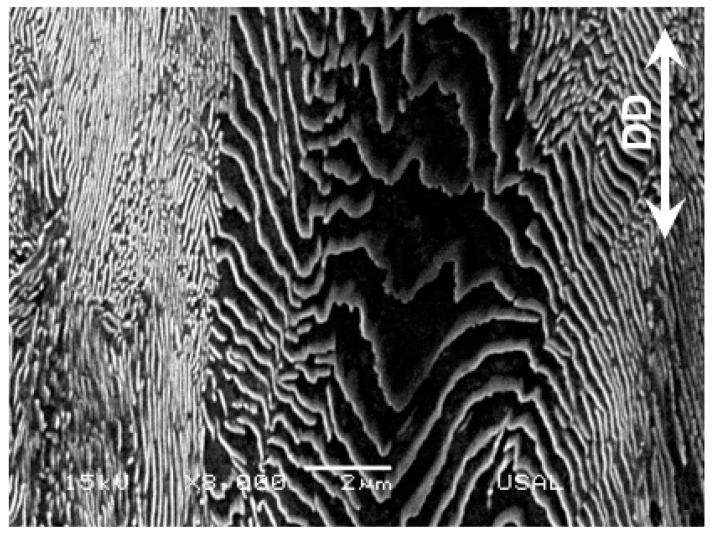
Pearlitic pseudocolony in a heavily cold-drawn pearlitic steel.

With regard to the environmentally assisted cracking of progressively cold-drawn pearlitic steels, the appearance of the so-called ‘pearlitic pseudocolony’ can be seen, cf. Figure 11 is responsible for crack path deflection in aggressive environments [56]. It is a sort of enlarged and oriented pearlitic colony inside which the lamellae are not properly oriented and then become curved and exhibiting an anomalous (extremely high) local interlamellar spacing, making it a weakest link or preferential fracture precursor promoting crack path deflection and thus, an anisotropic fracture.

## 5. Conclusions

The hierarchical microstructural evolution in pearlitic steels during cold drawing (at the two levels of pearlitic colonies and lamellae) shows the following trends: the slenderizing of the colonies, the decrease in interlamellar spacing and orientation in the direction of the cold drawing (wire axis) of both the colonies and the lamellae.

Apart from the aforesaid changes, cold drawing produces a series of damages at the finest microstructural level in cold-drawn pearlitic steels detected in three forms: (i) intercolonial tearing, (ii) multicolonial tearing, and (iii) micro-decolonization, the three contributing to different cracking mechanisms in the cold-drawn pearlitic steels.

The micro-decolonization could be responsible for the pop-in phenomenon in the case of fracture/cracking of heavily cold-drawn pearlitic steels, while intercolonial tearing could contribute to crack deflection and multicolonial tearing to the final phase of link up of micro-cracks, thereby producing a stepped crack path profile.

## Figures and Tables

**Table 1:** Chemical composition (wt.%) of steels of family E (the balance is Fe).

C	Mn	Si	P	S	Al	Cr	V
0.79	0.68	0.21	0.01	0.01	0.003	0.22	0.06

**Table 2:** Dimensions and mechanical properties of the steels analyzed.

Steel	*D* (mm)	*ε* ^P^_cum_	*E* (GPa)	*σ*_Y_ (GPa)	*σ*_R_ (GPa)	%*RA*
E0	11.03	0.00	199	0.72	1.23	31.24
E2	8.95	0.42	194	0.91	1.36	35.07
E4	7.49	0.78	196	1.02	1.50	41.67
E6	6.26	1.13	200	1.16	1.62	38.04
E7	5.04	1.57	208	1.49	1.83	23.05

## Data Availability

Not applicable.
